# ESforRPD2: Expert System for Rice Plant Disease Diagnosis

**DOI:** 10.12688/f1000research.16657.3

**Published:** 2024-07-11

**Authors:** Fahrul Agus, Muh. Ihsan, Dyna Marisa Khairina, Krishna Purnawan Candra

**Affiliations:** 1 GIS and Environment Modelling Lab. CSIT, Mulawarman University, Samarinda, East Kalimantan, 75242, Indonesia; 2 Department of Agricultural Product Technology, Faculty of Agriculture, Mulawarman University, Samarinda, 75123, Indonesia

**Keywords:** Expert System, Rice Plant Disease, Waterfall, Unified Modelling Language

## Abstract

One of the factors causing rice production disturbance in Indonesia is that farmers lack knowledge of early symptoms of rice plant diseases. These diseases are increasingly rampant because of the lack of experts. This study aimed to overcome this problem by providing an Expert System that helps farmers to make an early diagnosis of rice plant diseases.

Data of rice plant pests and diseases in 2016 were taken from Samarinda, East Kalimantan, Indonesia using an in-depth survey, and rice experts from the Department of Food Crops and Horticulture of East Kalimantan Province were recruited for the project. The Expert System for Rice Plant Disease Diagnosis, ESforRPD2, was developed based on the pest and disease experiences of the rice experts and uses a Waterfall Paradigm and Unified Modeling Language. This Expert System can detect 48 symptoms and 8 types of diseases of rice plants from 16 data tests with a sensitivity of 87.5%. The system can also provide recommendations for the treatment of identified diseases.

ESforRPD2 is available in Indonesian at http://esforrpd2.blog.unmul.ac.id

## Introduction

Correct diagnosis of symptoms in rice plant diseases, caused by bacteria, nematodes, fungi, phythoplasmal and viruses ^1– 4^, is very critical in supporting the productivity of rice plants. However, many regions in Indonesia have a huge problem because of a limited number of rice plant pathologists. The large plantation area of rice plants is also a problem due to logistical issues when visiting these sites, leading to difficulty obtaining disease evidence.

Along with other rapid technological developments, a technology known as Expert System (ES) ^5– 8^ has been developed to solve health ^9– 12^, education ^13^, business ^14^, and agriculture ^15, 16^, problems. ES is usually designed for a specific condition, i.e. variables of climate in cases of agriculture. This article proposes a new software based on ES for the diagnosis of disease in rice plants in the Samarinda region, Indonesia. Waterfall Paradigm was applied in designing this ES. The prototype, Expert System for Rice Plant Disease Diagnosis (ESforRPD2) is available at: http://esforrpd2.blog.unmul.ac.id.

## Methods

### Data collection and ES development

The ES of rice plant disease diagnosis was designed to help farmers and agricultural officials to diagnose rice plant diseases occurring in the Samarinda region, East Kalimantan province, Indonesia. Rice plant experts were recruited from the Seed Technology Development Division at the Department of Food Crops and Horticulture of East Kalimantan Province and from the Department of Agro-eco-technology of Agricultural Faculty of Mulawarman University (one expert from each). The experts were the primary source for information on rice plant symptoms and diseases. The two rice plant experts have experience in diagnosing rice plant disease in the region of East Kalimantan Province for 20 years. Symptoms and diseases specific for rice plant and their relationships (and their ranked importance) were derived from the experts by questionnaire (Supplementary File 1). This allows the ES to be specific for rice plant diseases diagnosis. This information was then used to construct the knowledge base for building the ES software.

The ES software was developed using the Waterfall paradigm as recommended by Sommerville ^17^ using five stages, i.e. (i) planning and requirement, (ii) analysis and software design, (iii) implementation and unit testing, (iv) integration and (v) system testing and operation and maintenance. ES architecture consists of three parts, namely the user interface, the inference engine and the knowledge base as proposed by Lucas and van der Gaag ^7^. The user interface is used as a consulting interface in order to obtain knowledge and advice from the ES, which would be like consulting an expert. In this ES, the inference engine works as a consultation system in processing input data to build a diagnosis based on the knowledge base developed. The construction of the knowledge-based system employs the Dempster-Shafer approach.

### Implementation

The implementation of the ESforRPD2 application is based on Unified Modelling Language ( Figure 1) as proposed by Sommerville ^17^, which consists of use case diagrams, activity diagrams, and class diagrams.

**Figure 1.**
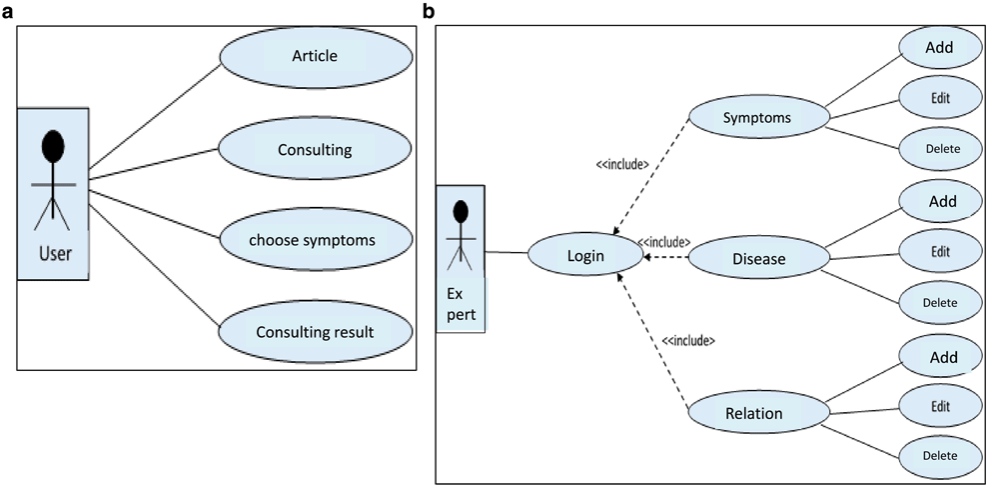
**a.** Use case diagram of user. **b.** Use case diagram of expert.

**Figure 1c.**
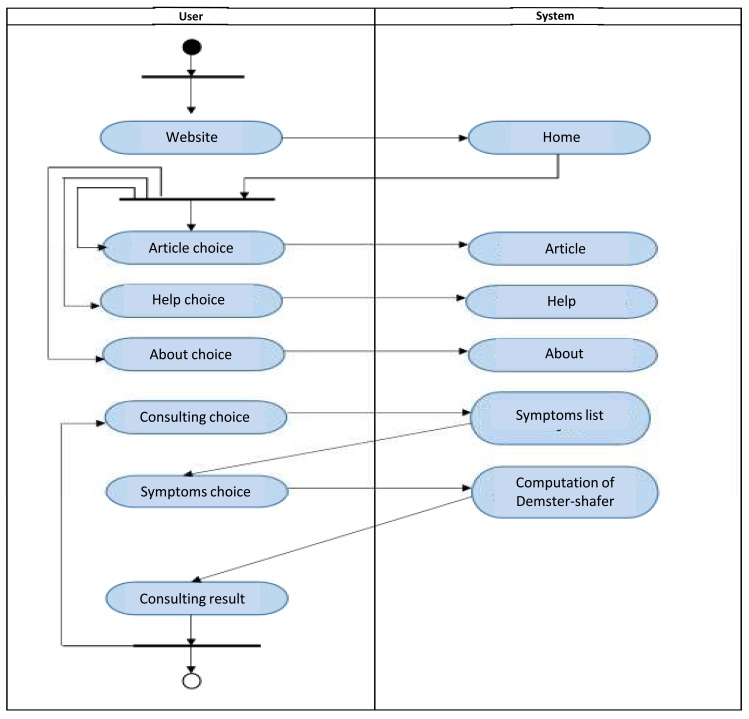
Activity diagram of ESforRPD2 system application.

**Figure 1d.**
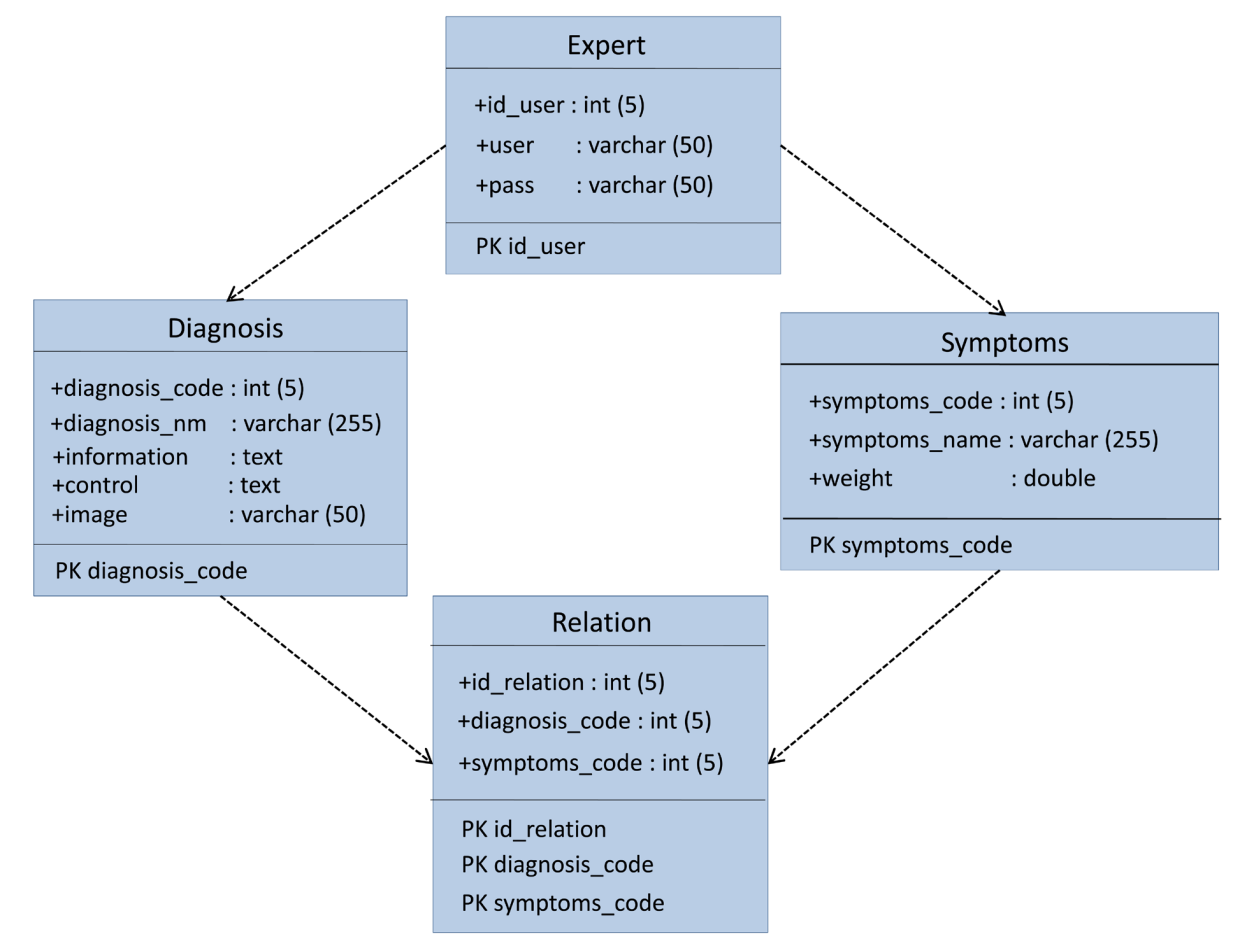
Class diagram of ESforRPD2 system application.

**Figure 2.**
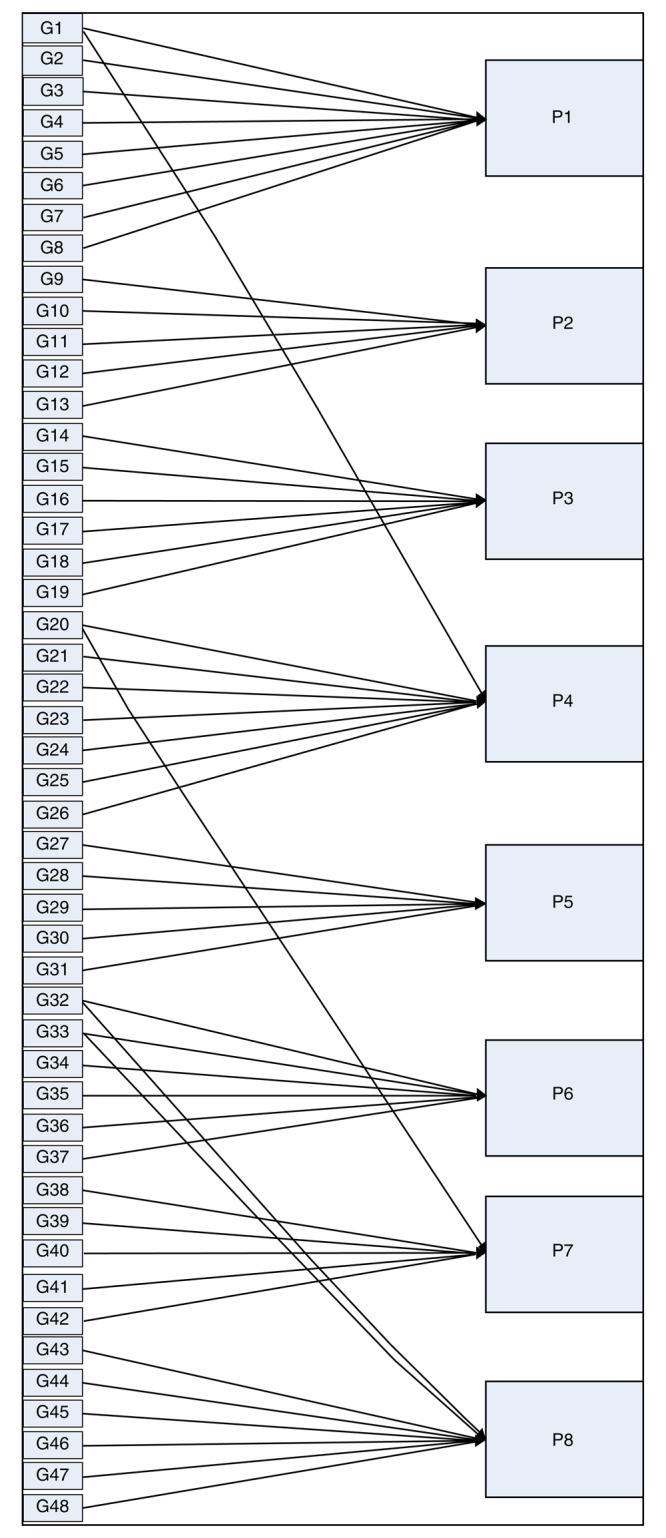
Decision tree and forward chaining tracing.

We constructed two types of “Use case diagram”, namely “Use case for user” consisting of four cases (Article, Consulting, Choose Symptoms and Consulting Result); and “Use case for expert” consisting of three cases (Symptoms, Diseases, and Relation). The use case describes the functions of the ES interacting with user and expert. The activity diagram illustrates the flow of various activities being designed in the ES, i.e. how the flow starts, the decision that might occur, and the flow end. The activity diagram also describes parallel processes that might occur in some executions. In this ES, we build four data stores (Expert, Symptoms, Relation, and Diagnosis) in the class diagram. The ESforRPD2 application uses four datasets, namely disease- and symptoms-data, knowledge base, and symptoms-disease-weight relationships table (Dataset 2). The construction of decision trees and forward-chaining tracing for diagnosing of rice plant diseases in the ES is shown in Figure 2.

ESforRPD2 is the first version of ES (in a bilingual interface that combines both English and Indonesia language) to make it user-friendly for Indonesian users. Users use a consultation page to choose the symptoms of the rice plant. The ES performs the calculation process to obtain the trust level using the Dempster-Shafer method ^18^. The user page ( Figure 3a) is the main web page for users without logging in. In the user page, there is also a home menu that displays articles about ES, rice plant diseases, and the Dempster-Shafer method. The consultation page starts the user consultation about the disease of rice plants ( Figure 3b). The ES will provide an output as a display showing the symptoms, diagnosis of disease and the confidence level ( Figure 3c).

**Figure 3a.**
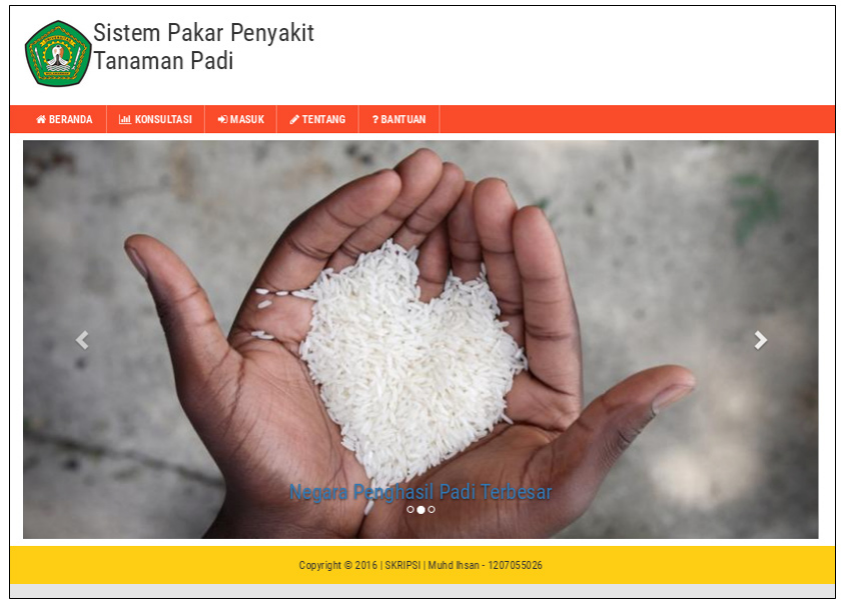
User main page.

**Figure 3b.**
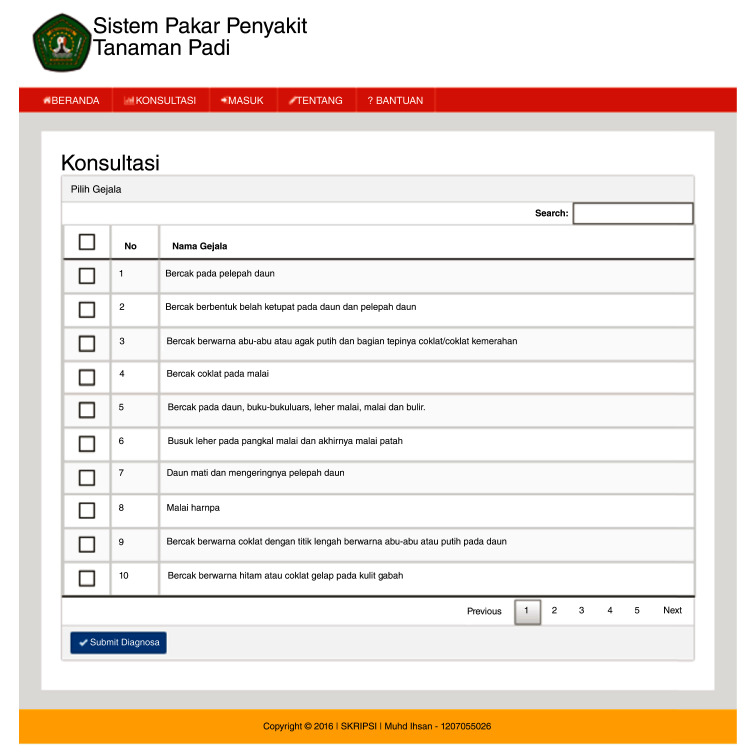
Consultation page.

**Figure 3c.**
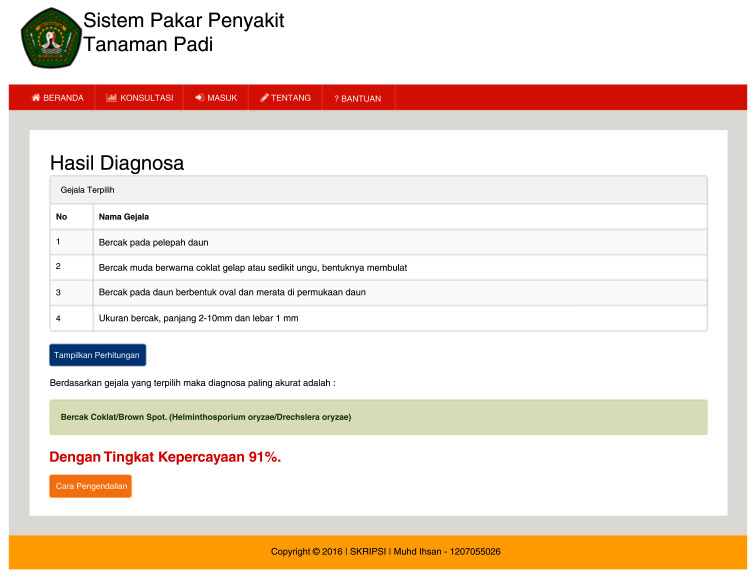
Diagnosis results page.

### Operation

The ESforRPD2 application is developed using CPU with specifications of Intel Core i3, 4GB RAM, and 300GB HDD. The same specification of CPU is needed to operate this application.

## Uses case

The ESforRPD2 application was tested applying symptom-data inputs by clicking the symptoms selected (Figure 5b). In a single test using the case of four symptom-data inputs selected, namely (i) Spots on leaf midrib, (ii) Little spots are dark brown or slightly purple rounded shape, (iii) Spots on oval-shaped leaves and evenly distributed on the leaf surface, (iv) The size of spots is 2–10 mm long and 1 mm wide, a display of diagnosis page ( Figure 3c) will appear following clicking of the “submit diagnose” button. The diagnosis page shows the confidence level. In this case test, the ES gave the sensitivity of disease type detection of 91%.

16 tests in row were conducted using randomly selected symptoms by user in the ES. The results were approved by the two experts. In total, 14 diagnosis (87.5%) of the 16 results showed by the ES were justified by the two experts ( Table 1).

**Table 1.** System testing with expert justification.

Test No.	Experts Justification (English/Indonesian)	Results Diagnosis of ESforRPD2 (English/Indonesian)	Results
1	Blast/Blas	Blast/Blast	Suitable
2	Brown Spot ( *Bercak Coklat*)	Brown Spot ( *Bercak Coklat*)	Suitable
3	Narrow Brown Spot ( *Bercak Coklat Sempit*)	Narrow Brown Spot ( *Bercak Coklat Sempit*)	Suitable
4	Sheath Bligh ( *Hawar Pelepah*)	Sheath Bligh ( *Hawar Pelepah*)	Suitable
5	False Smut ( *Noda Palsu/Gosong Palsu*)	False Smut ( *Noda Palsu/Gosong Palsu*)	Suitable
6	Grassy Stunt ( *Kerdil Rumput*)	Grassy Stunt ( *Kerdil Rumput*)	Suitable
7	Bacterial leaf blight ( *BLB-Kresek Hawar Daun*)	Bacterial leaf blight ( *BLB-Kresek Hawar Daun*)	Suitable
8	Tungro ( *Tungro*)	Tungro ( *Tungro*)	Suitable
9	Blast ( *Blas*)	Blast (Blas)	Suitable
10	Brown Spot ( *Bercak Coklat*)	Brown Spot ( *Bercak Coklat*)	Suitable
11	Narrow Brown Spot ( *Bercak Coklat Sempit*)	Blast/ *Blas*	Unsuitable
12	Sheath Bligh ( *Hawar Pelepah*)	Blast/ *Blas*	Unsuitable
13	False Smut ( *Noda Palsu/Gosong Palsu*)	False Smut ( *Noda Palsu/Gosong Palsu*)	Suitable
14	Grassy Stunt ( *Kerdil Rumput*)	Grassy Stunt ( *Kerdil Rumput*)	Suitable
15	Bacterial leaf blight ( *BLB-Kresek Hawar Daun*)	Bacterial leaf blight ( *BLB-Kresek Hawar Daun*)	Suitable
16	Tungro ( *Tungro*)	Tungro ( *Tungro*)	Suitable

## Discussion

The ESforRPD2 application is showing good reliability. By applying 16 tests, the ESforRPD2 showed a level of performance of 87.5% ( Table 1) following justification to two rice plants diseases experts. The performance of the ESforRPD2 during validation was the expected high-performance level of plant diseases diagnosis by the expert system. This performance is much higher than the performance of ES for Chili pepper pest diagnosis invented by Agus *et al.* ^16^. However other Expert System could show excellent performance of 98.38% ^19^, this evidence advice that the performance of ESforRPD2 could be improved in the next study.

Currently, ESforRPD2 has only been tested with data from the Samarinda region. In a future study, we will use data from other regions of East Kalimantan, which have the same climate (tropical rainforest) and soil character as the Samarinda region. In addition, we will test data from other regions in Indonesia, which have a different climate. Newbery *et al*. ^20^ showed that different climate conditions affect symptoms of arable crop disease; therefore, the ESforRPD2 will need continuous evaluation because climate change effects ^21^.

## Consent

Written informed consent was obtained from the two experts for participation in the study.

## Data Availability

Zenodo: Knowledge base for rice plant disease diagnosis, https://doi.org/10.5281/zenodo.1490658 ^23^ Zenodo: Dataset for rice plant diseases expert interview, http://doi.org/10.5281/zenodo.1953383 ^24^ Data are available under the terms of the CC BY 4.0 Attribution International License (CC BY 4.0)

## Software availability

Software application is available from: http://esforrpd2.blog.unmul.ac.id.

Source code: https://github.com/fahrulagus/paper.

Archived source code as at time of publication: https://doi.org/10.5281/zenodo.1490641 ^22^

License: GNU GPL v3.0
