# Supplementary material for: ESforRPD2: Expert System for Rice Plant Disease Diagnosis
Source: F1000Res. 2024 Jul 11;7:1902. Originally published 2018 Dec 6. [Version 3] doi: 10.12688/f1000research.16657.3 (PMC11325141; doi:10.12688/f1000research.16657.3)
Supplement: Peer review history

# Reviewer report: Reviewer response for version 3

**DOI:** 10.5256/f1000research.168667.r302069

**Published:** 2024-08-14

**Authors:** Marimin Marimin

**Affiliations:** 1 Department of Agricultural Industrial Technology, Faculty of Agricultural Technology, IPB University (Bogor Agricultural University), Bogor, Indonesia

I have read the newly revised version of the article. The authors have addressed all of my comments. Therefore I have no objection to approving this article to be indexed at the scopus.

Are the conclusions about the tool and its performance adequately supported by the findings presented in the article?

Partly

Is the rationale for developing the new software tool clearly explained?

Yes

Is the description of the software tool technically sound?

Yes

Are sufficient details of the code, methods and analysis (if applicable) provided to allow replication of the software development and its use by others?

Partly

Is sufficient information provided to allow interpretation of the expected output datasets and any results generated using the tool?

Partly

Reviewer Expertise:

NA

I confirm that I have read this submission and believe that I have an appropriate level of expertise to confirm that it is of an acceptable scientific standard.

# Reviewer report: Reviewer response for version 2

**DOI:** 10.5256/f1000research.19990.r59359

**Published:** 2020-05-22

**Authors:** Marimin Marimin

**Affiliations:** 1 Department of Agricultural Industrial Technology, Faculty of Agricultural Technology, IPB University (Bogor Agricultural University), Bogor, Indonesia

- In the last section of  abstract should be added that the system also provide suggestion for handling the identified disese.

- The interface of the implemented system is in Indonesian, It will be better beside the Indonesian, it should be available also in English.

- The data used is considered old, and only in one year, 2016. It will be better if new data were added and considered, such as in 2017, for better accuracy. Moreover, information on the experts contribute in the system development should be described, how many experts, expert qualification and the knowledge elicitation procedure should be described.

- In Expert system development there two concerned, software development and knowledge based development. Waterfall model used for software development is reasonably described. However, the knowledge based development is not described.
- The developed system used rube based which has drawback in maintenance difficulties due to the exponential grow of rules when some variables are added. Why not use fuzzy rule based or at least added in the discussion section for future enhancement?
- Discussion on how the disease are determined and the suggestion are provided should be added in the discussion section.

Are the conclusions about the tool and its performance adequately supported by the findings presented in the article?

Partly

Is the rationale for developing the new software tool clearly explained?

Yes

Is the description of the software tool technically sound?

Yes

Are sufficient details of the code, methods and analysis (if applicable) provided to allow replication of the software development and its use by others?

Partly

Is sufficient information provided to allow interpretation of the expected output datasets and any results generated using the tool?

Partly

Reviewer Expertise:

INtelligence decision support system and expert systems, Intelligence supply chain management.

I confirm that I have read this submission and believe that I have an appropriate level of expertise to confirm that it is of an acceptable scientific standard, however I have significant reservations, as outlined above.

# Response

**Published:** 2024-06-25

**Authors:** Fahrul Agus

**Affiliations:** Mulawarman University, Indonesia

**Reviewer 2:** In the last section of  abstract should be added that the system also provide suggestion for handling the identified disease.

**Response:** We appreciate your significant contribution. In the final section of the abstract, we will include further details, stating that the system also offers recommendations for the management of diagnosed illnesses.

**Reviewer 2: **The interface of the implemented system is in Indonesian, It will be better beside the Indonesian, it should be available also in English.

**Response: **Thank you for your valuable comments. We have developed a bilingual interface that combines both English and Indonesian languages. The functionalities are accessible in English, while the UI remains accessible in Indonesian. We developed this to improve accessibility for a wider demographic.

**Reviewer 2:** The data used is considered old, and only in one year, 2016. It will be better if new data were added and considered, such as in 2017, for better accuracy. Moreover, information on the experts contribute in the system development should be described, how many experts, expert qualifications, and the knowledge elicitation procedure.

**Response:** I appreciate your perceptive reply. We recognize the importance of integrating up-to-date data and will strive to incorporate 2017 data in order to improve precision. Despite the use of outdated data, this research remains relevant to identifying rice plant diseases.

**Reviewer 2:** In Expert system development there two concerned, software development and knowledge based development. Waterfall model used for software development is reasonably described. However, the knowledge based development is not described.

**Response:** I appreciate your valuable input. We value your acknowledgement of the software development process employing the waterfall approach. We have explained knowledge-based development by stating that the construction of this system utilizes the Dempster-Shafer technique.

**Reviewer 2:** The developed system used rube based which has drawback in maintenance difficulties due to the exponential grow of rules when some variables are added. Why not use fuzzy rule based or at least added in the discussion section for future enhancement?

**Response:** I appreciate your perceptive reply. We acknowledge the maintenance difficulties that arise from rule-based systems as a result of the rapid expansion of rules. We will take into account your recommendations to investigate the potential of fuzzy rule-based systems for future enhancements.

**Reviewer 2:** Discussion on how the disease are determined and the suggestion are provided should be added in the discussion section.

**Response: **I express my gratitude for your perceptive suggestion. In the sentence "The construction of decision trees and forward-chaining tracing for diagnosing rice plant diseases in the ES is shown in Figure 2," we have provided a comprehensive explanation of the process of identifying problems and providing the suggestion.

# Response

**Published:** 2024-06-25

**Authors:** Fahrul Agus

**Affiliations:** Mulawarman University, Indonesia

We appreciate your effort in reviewing our application and verifying its adherence to scientific standards. We highly appreciate your experience and greatly respect your input. We recognize the reservations you have specified and have addressed any feedback you have given. We also anticipate that you will cancel your reservation.

# Reviewer report: Reviewer response for version 2

**DOI:** 10.5256/f1000research.19990.r44749

**Published:** 2019-03-01

**Authors:** Yi Fang

**Affiliations:** 1 Nano Electrochemistry Laboratory, College of Engineering, University of Georgia, Athens, GA, USA

After the language revision and then using the correct terminologies, I agree that the manuscript should be indexed.

Are the conclusions about the tool and its performance adequately supported by the findings presented in the article?

Yes

Is the rationale for developing the new software tool clearly explained?

Partly

Is the description of the software tool technically sound?

Yes

Are sufficient details of the code, methods and analysis (if applicable) provided to allow replication of the software development and its use by others?

Yes

Is sufficient information provided to allow interpretation of the expected output datasets and any results generated using the tool?

Yes

Reviewer Expertise:

electrochemistry, plant diseases, biosensors, sensor

I confirm that I have read this submission and believe that I have an appropriate level of expertise to confirm that it is of an acceptable scientific standard.

# Reviewer report: Reviewer response for version 1

**DOI:** 10.5256/f1000research.18205.r43488

**Published:** 2019-01-24

**Authors:** Yi Fang

**Affiliations:** 1 Nano Electrochemistry Laboratory, College of Engineering, University of Georgia, Athens, GA, USA

The author applied Expert System (ES) for rice plant disease and diagnosis; the background information and introduction is sufficient and well organized. The entire manuscript is also presented well. However, the author used the word “accuracy” which is not quite a scientific term. If “accuracy” is defined as sensitivity of the method, how about the specificity of the method? If a method has low specificity, it may not be able to solve the problem from false positive. For other comments, please see below:

- Page 1: Change “is the lack of knowledge of farmers on early symptoms…” to “is that farmers lack of knowledge of early symptoms…”.
- Page 1: “accuracy of 87.5%” accuracy is not a scientific terminology, do you refer to sensitivity or specificity?
- Page 3: change “… education, and business, including agriculture, problems” to “…education, business, and agriculture problems.”
- Page 3: please change to “Waterfall Paradigm was applied in designing this ES.”
- Page 5: “In this case test, the ES gave the accuracy of disease type detection of 91%”. Do you refer to sensitivity? Please also try to apply this comment to the other “accuracy” you mentioned in the manuscript.

Are the conclusions about the tool and its performance adequately supported by the findings presented in the article?

Yes

Is the rationale for developing the new software tool clearly explained?

Partly

Is the description of the software tool technically sound?

Yes

Are sufficient details of the code, methods and analysis (if applicable) provided to allow replication of the software development and its use by others?

Yes

Is sufficient information provided to allow interpretation of the expected output datasets and any results generated using the tool?

Yes

Reviewer Expertise:

electrochemistry, plant diseases, biosensors, sensor

I confirm that I have read this submission and believe that I have an appropriate level of expertise to confirm that it is of an acceptable scientific standard, however I have significant reservations, as outlined above.

# Response

**Published:** 2019-02-12

**Authors:** Fahrul Agus

**Affiliations:** Mulawarman University, Indonesia

We agree with your judgment regarding the term of accuracy. We meant the accuracy is the sensitivity, for that reason we change the term accuracy to sensitivity. Regarding the term of specificity, we explain that this system has high specificity for rice plants because all data used in constructing the algorithm were collected specifically for rice plant diseases.
